# *catena*-Poly[bis­(μ_3_-2-methyl­benzoato)disilver(I)]

**DOI:** 10.1107/S1600536811016801

**Published:** 2011-05-07

**Authors:** Muhammad Danish, M. Nawaz Tahir, Sabiha Ghafoor, Nazir Ahmad, Mehwish Nisa

**Affiliations:** a Department of Chemistry, University of Gujrat, Department of Chemistry, Hafiz Hayat Campus, Gujrat, Pakistan; b Department of Physics, University of Sargodha, Sargodha, Pakistan; c Department of Chemistry, University of Sargodha, Sargodha, Pakistan

## Abstract

The crystal structure of the title compound, [Ag_2_(C_8_H_7_O_2_)_2_]_*n*_, features polymeric chains extending along the *a* axis, with the two Ag^+^ cations in a distorted trigonal coordination. The range of Ag—O bond lengths is 2.169 (2)–2.433 (2) Å, whereas the Ag⋯Ag separations are in the range 2.8674 (4)–3.6256 (5) Å. The 2-methyl­benzoate groups are oriented at a dihedral angle of 60.7 (1)° with respect to each other.

## Related literature

For metal complexes of *o*-toluic acid, see: Danish *et al.* (2010**a* ▶,*b* ▶,c* ▶). For the crystal structures of related silver complexes, see: Tahir *et al.* (1996 ▶, 2009 ▶); Ülkü *et al.* (1996 ▶).
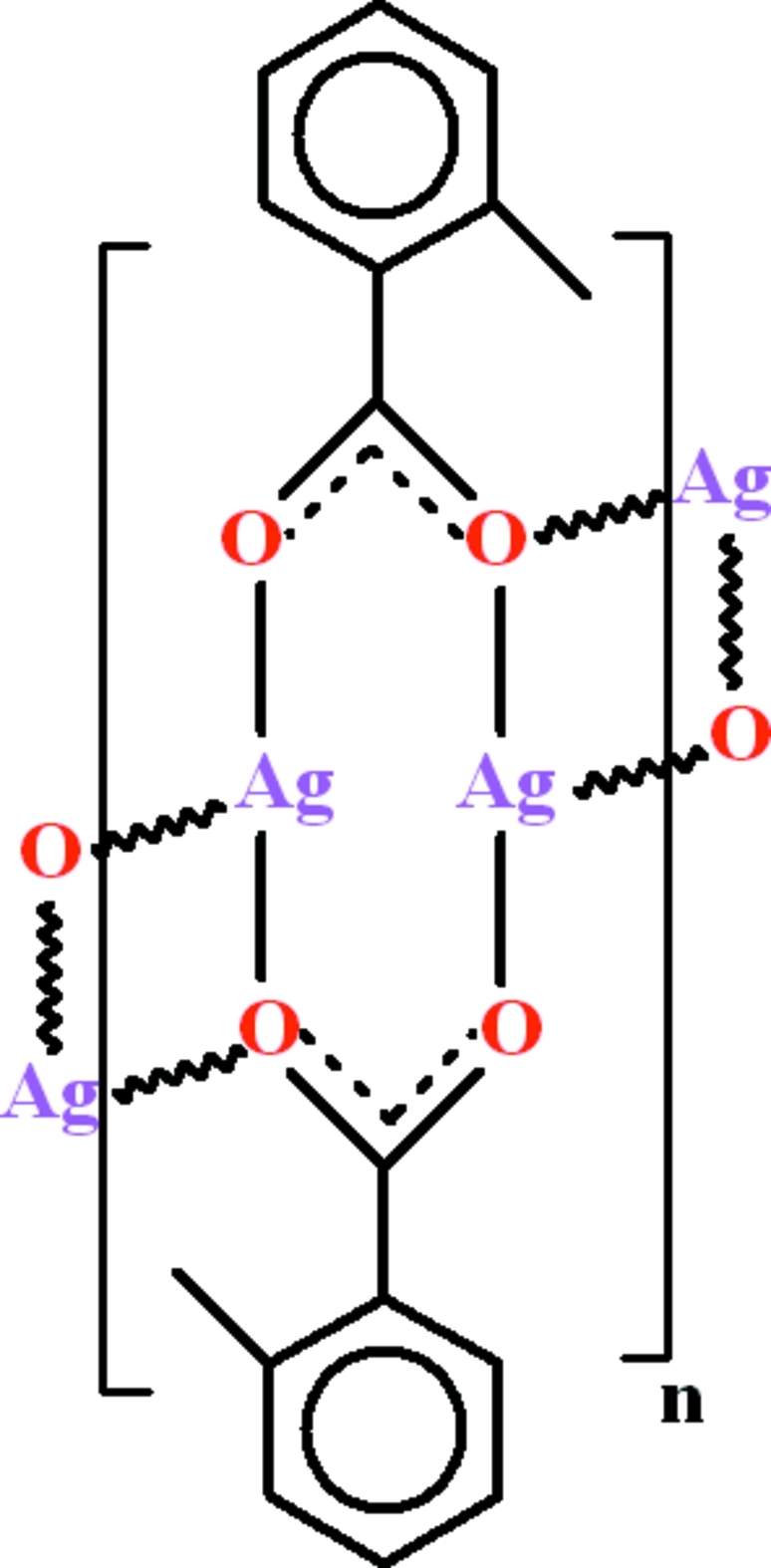


## Experimental

### 

#### Crystal data

[Ag_2_(C_8_H_7_O_2_)_2_]
*M* *_r_* = 486.01
Monoclinic, 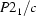

*a* = 5.6607 (3) Å
*b* = 27.1493 (18) Å
*c* = 10.2455 (7) Å
β = 100.538 (3)°
*V* = 1548.01 (17) Å^3^
*Z* = 4
Mo *K*α radiation
μ = 2.54 mm^−1^
*T* = 296 K
0.28 × 0.15 × 0.13 mm

#### Data collection

Bruker Kappa APEXII CCD diffractometer
Absorption correction: multi-scan (*SADABS*; Bruker, 2005 ▶) *T*_min_ = 0.465, *T*_max_ = 0.555
12447 measured reflections
3486 independent reflections
2487 reflections with *I* > 2σ(*I*)
*R*_int_ = 0.036

#### Refinement

*R*[*F* ^2^ > 2σ(*F* ^2^)] = 0.034
*wR*(*F* ^2^) = 0.061
*S* = 1.04
3486 reflections
201 parameters
H-atom parameters constrained
Δρ_max_ = 0.44 e Å^−3^
Δρ_min_ = −0.54 e Å^−3^

### 

Data collection: *APEX2* (Bruker, 2009 ▶); cell refinement: *SAINT* (Bruker, 2009 ▶); data reduction: *SAINT*; program(s) used to solve structure: *SHELXS97* (Sheldrick, 2008 ▶); program(s) used to refine structure: *SHELXL97* (Sheldrick, 2008 ▶); molecular graphics: *ORTEP-3 for Windows* (Farrugia, 1997 ▶) and *PLATON* (Spek, 2009 ▶); software used to prepare material for publication: *WinGX* (Farrugia, 1999 ▶) and *PLATON*.

## Supplementary Material

Crystal structure: contains datablocks global, I. DOI: 10.1107/S1600536811016801/ez2241sup1.cif

Structure factors: contains datablocks I. DOI: 10.1107/S1600536811016801/ez2241Isup2.hkl

Additional supplementary materials:  crystallographic information; 3D view; checkCIF report

## Figures and Tables

**Table:** 

Ag1—O1	2.433 (2)
Ag1—O3	2.268 (2)
Ag1—O2^i^	2.169 (2)
Ag2—O1	2.305 (2)
Ag2—O3	2.414 (2)
Ag2—O4^ii^	2.186 (3)

**Table:** 

O1—Ag1—O3	79.34 (8)
O1—Ag1—O2^i^	121.02 (8)
O2^i^—Ag1—O3	153.38 (10)
O1—Ag2—O3	79.04 (8)
O1—Ag2—O4^ii^	149.80 (9)
O3—Ag2—O4^ii^	122.08 (9)
Ag1—O1—Ag2	99.83 (9)
Ag1—O3—Ag2	101.47 (9)

Symmetry codes: (i) 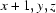; (ii) 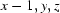.

**Fig. 1.**
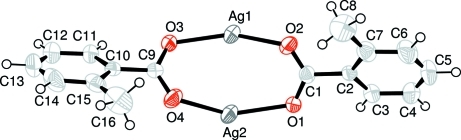
View of the title compound with the atom numbering scheme. The thermal ellipsoids are drawn at the 50% probability level.

**Fig. 2.**
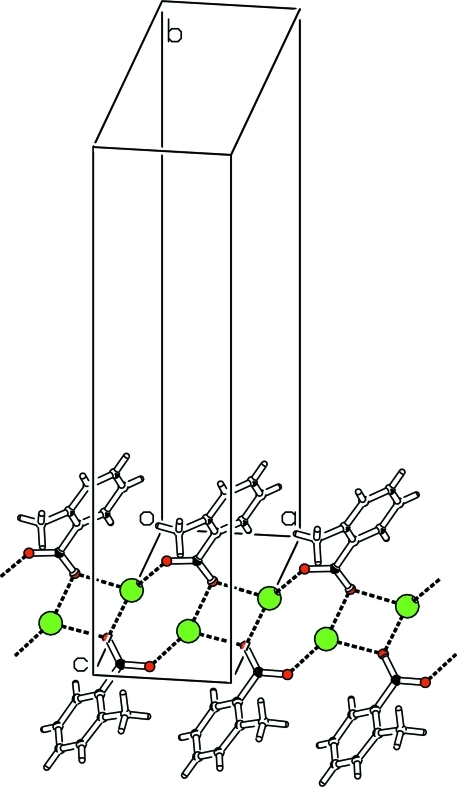
A partial packing diagram (PLATON; Spek, 2009) which shows that the molecules are interlinked to form polymeric chains.

**Fig. 3.**
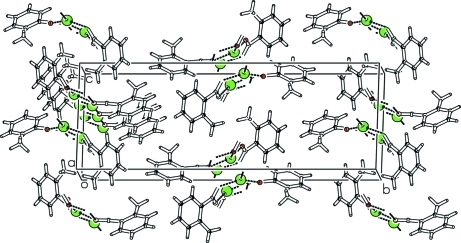
A partial packing diagram (PLATON; Spek, 2009) which shows that the polymeric chains are linked into pairs.

## supplementary crystallographic information

### Comment

The title compound (I, Fig. 1) is a continuation of our work on the synthesis of metal complexes of *o*-toluic acid, where we have reported the crystal structures of (II), *catena*-poly[[trimethyltin(IV)]-µ-2-methylbenzoato-κ^2^*O*:*O*^'^] (Danish *et al.*, 2010*a*), (III), tetrakis(-2-methylbenzoato-κ^2^*O*:*O*^'^)bis[(methanol-κ*O*)copper(II)] (Danish *et al.*, 2010*b*) and (IV), octamethylbis(2–2-methylbenzoato-κ^2^*O*:*O*^'^)bis(2-methylbenzoato-κ*O*) di-µ_3_-oxido-tetratin(IV) (Danish *et al.*, 2010*c*).

We have also reported the crystal structures of silver complexes such as (V), poly[bis(*p*-nitrosalicylato-O:*O*^'^)disilver(I)—O^3^:Ag';Ag:*O*^3'^] (Tahir *et al.*, 1996), (VI), poly[bis(3,5-dinitrobenzoato-O^1^:*O*^2^)disilver(I)—O^2^:Ag;Ag^'^:*O*^2'^] (Ülkü *et al.*, 1996) and (VII), poly[(µ-benzene-1,2,4,5-tetracarboxylato)tetrasilver(I)] (Tahir *et al.*, 2009).

In the title compound, the toluine groups A (C2—C8) and B (C10—C16) are planar with r.m.s. deviation of 0.0063 and 0.0086 Å. The carboxylate groups C (O1/C1/O2) and D (O3/C9/O4) are of course planar with dihedral angles between A/C, B/D & A/B of 34.6 (3)°, 37.5 (3)° and 60.7 (1)°, respectively. The title compound essentially consists of non-centrosymmetric dimers with central core E (Ag1/O2/C1/O1/Ag2/O4/C9/O3) which is not planar (Fig. 1). These dimers are interlinked via Ag—O bonds to form one-dimensional polymeric chains extending along the *a*-axis (Fig. 2). The parallel polymeric chains are further interlinked by Ag—O bonds into dimeric polymeric chains. In the central core the range of Ag—O bond distances is 2.268 (2)–2.433 (2) Å whereas to adjacent units they are 2.169 (2) and 2.186 (3) Å. The Ag···Ag distance for the central core is 2.8674 (4) Å, whereas it is 3.6256 (5) Å for the symmetry related adjacent units forming the four membered ring F (Ag_2_O_2_). The Ag···Ag separations for adjacent chains are 3.1292 (6) and 3.2314 (6) Å. The important bond distances and angles are given in Table 1.

### Experimental

Aqueous solutions of silver nitrate (0.17 g, 1.0 mmol) and the sodium salt of *o*-toluic acid (0.122 g, 1.0 mmol) were prepared separately in 5.0 and 10.0 ml of water, respectively. The aqueous silver nitrate was added dropwise to the solution of the sodium salt of *o*-toluic acid with continuous stirring until a white precipitate appeared. The reaction mixture was filtered after treatment with liquid ammonia. It was concentrated and kept in the dark for crystallization. White needle-like crystals appeared within two months.

Melting point: 473 K.

### Refinement

The H-atoms were positioned geometrically (C–H = 0.93–0.96 Å) and refined as riding with *U*_iso_(H) = *xU*_eq_(C), where *x* = 1.5 for methyl and *x* = 1.2 for aryl H-atoms.

### Crystal data

**Table:**

[Ag_2_(C_8_H_7_O_2_)_2_]	*F*(000) = 944
*M**_r_* = 486.01	*D*_x_ = 2.085 Mg m^−^^3^
Monoclinic, *P*2_1_/*c*	Mo *K*α radiation, λ = 0.71073 Å
Hall symbol: -P 2ybc	Cell parameters from 2487 reflections
*a* = 5.6607 (3) Å	θ = 3.0–27.6°
*b* = 27.1493 (18) Å	µ = 2.54 mm^−^^1^
*c* = 10.2455 (7) Å	*T* = 296 K
β = 100.538 (3)°	Needle, colorless
*V* = 1548.01 (17) Å^3^	0.28 × 0.15 × 0.13 mm
*Z* = 4	

### Data collection

**Table:**

Bruker Kappa APEXII CCD diffractometer	3486 independent reflections
Radiation source: fine-focus sealed tube	2487 reflections with *I* > 2σ(*I*)
graphite	*R*_int_ = 0.036
Detector resolution: 7.60 pixels mm^-1^	θ_max_ = 27.6°, θ_min_ = 3.0°
ω scans	*h* = −6→7
Absorption correction: multi-scan (*SADABS*; Bruker, 2005)	*k* = −30→35
*T*_min_ = 0.465, *T*_max_ = 0.555	*l* = −13→13
12447 measured reflections	

### Refinement

**Table:**

Refinement on *F*^2^	Primary atom site location: structure-invariant direct methods
Least-squares matrix: full	Secondary atom site location: difference Fourier map
*R*[*F*^2^ > 2σ(*F*^2^)] = 0.034	Hydrogen site location: inferred from neighbouring sites
*wR*(*F*^2^) = 0.061	H-atom parameters constrained
*S* = 1.04	*w* = 1/[σ^2^(*F*_o_^2^) + (0.0203*P*)^2^] where *P* = (*F*_o_^2^ + 2*F*_c_^2^)/3
3486 reflections	(Δ/σ)_max_ = 0.001
201 parameters	Δρ_max_ = 0.44 e Å^−^^3^
0 restraints	Δρ_min_ = −0.54 e Å^−^^3^

### Special details

**Table:**

Geometry. Bond distances, angles *etc*. have been calculated using the rounded fractional coordinates. All su's are estimated from the variances of the (full) variance-covariance matrix. The cell e.s.d.'s are taken into account in the estimation of distances, angles and torsion angles
Refinement. Refinement of *F*^2^ against ALL reflections. The weighted *R*-factor *wR* and goodness of fit *S* are based on *F*^2^, conventional *R*-factors *R* are based on *F*, with *F* set to zero for negative *F*^2^. The threshold expression of *F*^2^ > σ(*F*^2^) is used only for calculating *R*-factors(gt) *etc*. and is not relevant to the choice of reflections for refinement. *R*-factors based on *F*^2^ are statistically about twice as large as those based on *F*, and *R*- factors based on ALL data will be even larger.

### Fractional atomic coordinates and isotropic or equivalent isotropic displacement parameters (Å^2^)

**Table:**

	*x*	*y*	*z*	*U*_iso_*/*U*_eq_	
Ag1	1.07402 (5)	0.04830 (1)	0.59037 (3)	0.0523 (1)	
Ag2	0.51817 (5)	−0.00478 (1)	0.65370 (3)	0.0479 (1)	
O1	0.6443 (4)	0.06445 (8)	0.5558 (3)	0.0400 (9)	
O2	0.3299 (4)	0.10785 (8)	0.5914 (3)	0.0575 (12)	
O3	0.9407 (4)	−0.02228 (8)	0.6706 (3)	0.0422 (9)	
O4	1.3029 (4)	−0.03844 (9)	0.7865 (3)	0.0552 (10)	
C1	0.5350 (6)	0.10473 (12)	0.5654 (3)	0.0377 (12)	
C2	0.6596 (5)	0.15172 (11)	0.5403 (3)	0.0342 (11)	
C3	0.8070 (6)	0.15067 (13)	0.4455 (4)	0.0464 (14)	
C4	0.9228 (7)	0.19266 (16)	0.4135 (4)	0.0632 (17)	
C5	0.8928 (8)	0.23558 (16)	0.4780 (6)	0.075 (2)	
C6	0.7493 (7)	0.23710 (14)	0.5728 (5)	0.0678 (18)	
C7	0.6290 (6)	0.19559 (13)	0.6056 (4)	0.0496 (14)	
C8	0.4769 (7)	0.19959 (15)	0.7121 (5)	0.078 (2)	
C9	1.0825 (6)	−0.04380 (12)	0.7643 (4)	0.0347 (12)	
C10	0.9718 (6)	−0.07847 (12)	0.8498 (4)	0.0362 (11)	
C11	0.7793 (6)	−0.10729 (12)	0.7884 (4)	0.0491 (14)	
C12	0.6741 (7)	−0.14127 (14)	0.8585 (5)	0.0678 (19)	
C13	0.7550 (8)	−0.14592 (16)	0.9913 (6)	0.076 (2)	
C14	0.9405 (9)	−0.11792 (16)	1.0530 (5)	0.0698 (19)	
C15	1.0591 (7)	−0.08369 (13)	0.9848 (4)	0.0482 (16)	
C16	1.2646 (8)	−0.05403 (15)	1.0582 (5)	0.0762 (19)	
H3	0.82809	0.12119	0.40290	0.0558*	
H4	1.01920	0.19166	0.34913	0.0756*	
H5	0.97014	0.26403	0.45779	0.0903*	
H6	0.73227	0.26667	0.61591	0.0815*	
H8A	0.51961	0.17373	0.77593	0.1167*	
H8B	0.31020	0.19663	0.67235	0.1167*	
H8C	0.50419	0.23095	0.75550	0.1167*	
H11	0.72115	−0.10338	0.69809	0.0586*	
H12	0.54878	−0.16090	0.81582	0.0812*	
H13	0.68275	−0.16843	1.04006	0.0910*	
H14	0.99129	−0.12151	1.14409	0.0837*	
H16A	1.23586	−0.01969	1.03935	0.1139*	
H16B	1.41064	−0.06385	1.03049	0.1139*	
H16C	1.27876	−0.05956	1.15189	0.1139*	

### Atomic displacement parameters (Å^2^)

**Table:**

	*U*^11^	*U*^22^	*U*^33^	*U*^12^	*U*^13^	*U*^23^
Ag1	0.0348 (2)	0.0527 (2)	0.0712 (3)	−0.0063 (1)	0.0144 (2)	0.0106 (2)
Ag2	0.0333 (2)	0.0541 (2)	0.0570 (2)	−0.0083 (1)	0.0100 (1)	0.0043 (2)
O1	0.0319 (13)	0.0331 (13)	0.0546 (18)	−0.0010 (10)	0.0068 (11)	0.0059 (11)
O2	0.0373 (15)	0.0417 (15)	0.098 (3)	−0.0036 (11)	0.0243 (14)	0.0008 (14)
O3	0.0315 (13)	0.0450 (14)	0.0498 (18)	−0.0013 (11)	0.0068 (12)	0.0130 (12)
O4	0.0298 (14)	0.0739 (19)	0.060 (2)	−0.0071 (12)	0.0036 (12)	0.0187 (14)
C1	0.038 (2)	0.037 (2)	0.036 (2)	−0.0049 (16)	0.0015 (16)	0.0030 (16)
C2	0.0287 (18)	0.0309 (19)	0.040 (2)	−0.0002 (14)	−0.0016 (15)	0.0043 (16)
C3	0.046 (2)	0.040 (2)	0.053 (3)	−0.0019 (17)	0.0085 (18)	0.0061 (19)
C4	0.058 (3)	0.061 (3)	0.074 (3)	−0.003 (2)	0.021 (2)	0.022 (2)
C5	0.073 (3)	0.043 (3)	0.110 (5)	−0.018 (2)	0.016 (3)	0.015 (3)
C6	0.074 (3)	0.037 (2)	0.089 (4)	−0.007 (2)	0.006 (3)	−0.007 (2)
C7	0.053 (2)	0.040 (2)	0.053 (3)	−0.0025 (18)	0.002 (2)	−0.0024 (19)
C8	0.093 (4)	0.072 (3)	0.073 (4)	−0.004 (3)	0.028 (3)	−0.027 (3)
C9	0.032 (2)	0.035 (2)	0.037 (2)	0.0027 (16)	0.0064 (16)	0.0004 (16)
C10	0.0333 (19)	0.036 (2)	0.041 (2)	0.0093 (16)	0.0114 (16)	0.0070 (17)
C11	0.043 (2)	0.043 (2)	0.062 (3)	−0.0039 (18)	0.0111 (19)	0.011 (2)
C12	0.057 (3)	0.054 (3)	0.095 (4)	−0.006 (2)	0.021 (3)	0.019 (3)
C13	0.080 (4)	0.062 (3)	0.097 (5)	0.005 (3)	0.044 (3)	0.032 (3)
C14	0.099 (4)	0.069 (3)	0.047 (3)	0.026 (3)	0.028 (3)	0.026 (2)
C15	0.062 (3)	0.043 (2)	0.041 (3)	0.0166 (19)	0.013 (2)	0.0033 (19)
C16	0.102 (4)	0.081 (3)	0.040 (3)	0.007 (3)	−0.002 (3)	−0.007 (2)

### Geometric parameters (Å, °)

**Table:**

Ag1—O1	2.433 (2)	C10—C15	1.388 (6)
Ag1—O3	2.268 (2)	C11—C12	1.370 (6)
Ag1—O2^i^	2.169 (2)	C12—C13	1.360 (8)
Ag2—O1	2.305 (2)	C13—C14	1.356 (7)
Ag2—O3	2.414 (2)	C14—C15	1.405 (6)
Ag2—O4^ii^	2.186 (3)	C15—C16	1.499 (6)
O1—C1	1.269 (4)	C3—H3	0.9300
O2—C1	1.241 (4)	C4—H4	0.9300
O3—C9	1.275 (5)	C5—H5	0.9300
O4—C9	1.235 (4)	C6—H6	0.9300
C1—C2	1.503 (4)	C8—H8A	0.9600
C2—C3	1.392 (5)	C8—H8B	0.9600
C2—C7	1.392 (5)	C8—H8C	0.9600
C3—C4	1.384 (6)	C11—H11	0.9300
C4—C5	1.365 (6)	C12—H12	0.9300
C5—C6	1.376 (7)	C13—H13	0.9300
C6—C7	1.389 (5)	C14—H14	0.9300
C7—C8	1.512 (6)	C16—H16A	0.9600
C9—C10	1.499 (5)	C16—H16B	0.9600
C10—C11	1.395 (5)	C16—H16C	0.9600
			
O1—Ag1—O3	79.34 (8)	C11—C12—C13	119.2 (4)
O1—Ag1—O2^i^	121.02 (8)	C12—C13—C14	120.3 (5)
O2^i^—Ag1—O3	153.38 (10)	C13—C14—C15	122.6 (5)
O1—Ag2—O3	79.04 (8)	C10—C15—C14	116.6 (4)
O1—Ag2—O4^ii^	149.80 (9)	C10—C15—C16	123.2 (4)
O3—Ag2—O4^ii^	122.08 (9)	C14—C15—C16	120.2 (4)
Ag1—O1—Ag2	99.83 (9)	C2—C3—H3	119.00
Ag1—O1—C1	129.2 (2)	C4—C3—H3	119.00
Ag2—O1—C1	118.4 (2)	C3—C4—H4	121.00
Ag1^ii^—O2—C1	126.8 (2)	C5—C4—H4	121.00
Ag1—O3—Ag2	101.47 (9)	C4—C5—H5	120.00
Ag1—O3—C9	117.0 (2)	C6—C5—H5	120.00
Ag2—O3—C9	128.6 (2)	C5—C6—H6	119.00
Ag2^i^—O4—C9	126.4 (3)	C7—C6—H6	119.00
O1—C1—O2	124.3 (3)	C7—C8—H8A	109.00
O1—C1—C2	117.9 (3)	C7—C8—H8B	109.00
O2—C1—C2	117.8 (3)	C7—C8—H8C	109.00
C1—C2—C3	117.5 (3)	H8A—C8—H8B	109.00
C1—C2—C7	122.7 (3)	H8A—C8—H8C	110.00
C3—C2—C7	119.7 (3)	H8B—C8—H8C	109.00
C2—C3—C4	121.3 (3)	C10—C11—H11	119.00
C3—C4—C5	118.8 (4)	C12—C11—H11	119.00
C4—C5—C6	120.6 (4)	C11—C12—H12	120.00
C5—C6—C7	121.7 (4)	C13—C12—H12	120.00
C2—C7—C6	117.9 (3)	C12—C13—H13	120.00
C2—C7—C8	123.1 (3)	C14—C13—H13	120.00
C6—C7—C8	119.0 (3)	C13—C14—H14	119.00
O3—C9—O4	124.1 (3)	C15—C14—H14	119.00
O3—C9—C10	117.1 (3)	C15—C16—H16A	109.00
O4—C9—C10	118.9 (3)	C15—C16—H16B	109.00
C9—C10—C11	117.7 (3)	C15—C16—H16C	109.00
C9—C10—C15	122.4 (3)	H16A—C16—H16B	109.00
C11—C10—C15	119.9 (3)	H16A—C16—H16C	109.00
C10—C11—C12	121.3 (4)	H16B—C16—H16C	109.00
			
O3—Ag1—O1—Ag2	4.46 (10)	Ag2^i^—O4—C9—C10	164.8 (2)
O3—Ag1—O1—C1	144.3 (3)	O1—C1—C2—C3	33.9 (4)
O2^i^—Ag1—O1—Ag2	−157.03 (10)	O1—C1—C2—C7	−147.5 (3)
O2^i^—Ag1—O1—C1	−17.2 (3)	O2—C1—C2—C3	−144.7 (3)
O1—Ag1—O3—Ag2	−4.28 (10)	O2—C1—C2—C7	34.0 (5)
O1—Ag1—O3—C9	−149.1 (3)	C1—C2—C3—C4	178.1 (3)
O2^i^—Ag1—O3—Ag2	138.32 (17)	C7—C2—C3—C4	−0.7 (5)
O2^i^—Ag1—O3—C9	−6.5 (4)	C1—C2—C7—C6	−178.7 (3)
O1—Ag1—O2^i^—C1^i^	−155.1 (3)	C1—C2—C7—C8	2.9 (5)
O3—Ag1—O2^i^—C1^i^	69.1 (4)	C3—C2—C7—C6	−0.1 (5)
O3—Ag2—O1—Ag1	−4.20 (10)	C3—C2—C7—C8	−178.5 (4)
O3—Ag2—O1—C1	−149.6 (3)	C2—C3—C4—C5	0.8 (6)
O4^ii^—Ag2—O1—Ag1	133.77 (16)	C3—C4—C5—C6	−0.2 (7)
O4^ii^—Ag2—O1—C1	−11.6 (4)	C4—C5—C6—C7	−0.5 (7)
O1—Ag2—O3—Ag1	4.53 (11)	C5—C6—C7—C2	0.7 (6)
O1—Ag2—O3—C9	143.6 (3)	C5—C6—C7—C8	179.1 (4)
O4^ii^—Ag2—O3—Ag1	−152.06 (10)	O3—C9—C10—C11	37.4 (5)
O4^ii^—Ag2—O3—C9	−13.0 (3)	O3—C9—C10—C15	−144.7 (4)
O1—Ag2—O4^ii^—C9^ii^	73.6 (4)	O4—C9—C10—C11	−141.2 (4)
O3—Ag2—O4^ii^—C9^ii^	−157.3 (3)	O4—C9—C10—C15	36.7 (5)
Ag1—O1—C1—O2	−153.9 (3)	C9—C10—C11—C12	177.4 (3)
Ag1—O1—C1—C2	27.7 (4)	C15—C10—C11—C12	−0.5 (5)
Ag2—O1—C1—O2	−20.2 (4)	C9—C10—C15—C14	−179.2 (4)
Ag2—O1—C1—C2	161.5 (2)	C9—C10—C15—C16	2.4 (6)
Ag1^ii^—O2—C1—O1	−12.7 (5)	C11—C10—C15—C14	−1.4 (5)
Ag1^ii^—O2—C1—C2	165.7 (2)	C11—C10—C15—C16	−179.8 (3)
Ag1—O3—C9—O4	−22.9 (5)	C10—C11—C12—C13	1.9 (6)
Ag1—O3—C9—C10	158.5 (2)	C11—C12—C13—C14	−1.3 (7)
Ag2—O3—C9—O4	−156.8 (3)	C12—C13—C14—C15	−0.8 (7)
Ag2—O3—C9—C10	24.7 (4)	C13—C14—C15—C10	2.1 (6)
Ag2^i^—O4—C9—O3	−13.8 (5)	C13—C14—C15—C16	−179.5 (4)

Symmetry codes: (i) *x*+1, *y*, *z*; (ii) *x*−1, *y*, *z*.
